# Gemcitabine-Loaded Magnetically Responsive Poly(*ε*-caprolactone) Nanoparticles against Breast Cancer

**DOI:** 10.3390/polym12122790

**Published:** 2020-11-25

**Authors:** Gracia García-García, Fátima Fernández-Álvarez, Laura Cabeza, Ángel V. Delgado, Consolación Melguizo, José C. Prados, José L. Arias

**Affiliations:** 1 Department of Pharmacy and Pharmaceutical Technology, Faculty of Pharmacy, University of Granada, 18071 Granada, Spain; graciagg3@gmail.com (G.G.-G.); fatimaferal@correo.ugr.es (F.F.-Á.); 2 Faculty of Experimental Sciences, Universidad Francisco de Vitoria, 28223 Madrid, Spain; 3 Institute of Biopathology and Regenerative Medicine (IBIMER), Center of Biomedical Research (CIBM), University of Granada, 18100 Granada, Spain; lautea@ugr.es (L.C.); melguizo@ugr.es (C.M.); jcprados@ugr.es (J.C.P.); 4 Biosanitary Research Institute of Granada (ibs.GRANADA), Andalusian Health Service (SAS), University of Granada, 18100 Granada, Spain; 5 Department of Human Anatomy and Embryology, Faculty of Medicine, University of Granada, 18016 Granada, Spain; 6 Department of Applied Physics, Faculty of Sciences, University of Granada, 18071 Granada, Spain; adelgado@ugr.es

**Keywords:** breast cancer, core/shell, drug loading, Gemcitabine, magnetic drug delivery, magnetite, pH-responsive drug release, poly(*ε*-caprolactone), polymer-coated nanoparticle

## Abstract

A reproducible and efficient interfacial polymer disposition method has been used to formulate magnetite/poly(*ε*-caprolactone) (core/shell) nanoparticles (average size ≈ 125 nm, production performance ≈ 90%). To demonstrate that the iron oxide nuclei were satisfactorily embedded within the polymeric solid matrix, a complete analysis of these nanocomposites by, e.g., electron microscopy visualizations, energy dispersive X-ray spectroscopy, Fourier-transform infrared spectroscopy, electrophoresis, and contact angle goniometry was conducted. The magnetic responsive behaviour of these nanoparticles was quantitatively characterized by the hysteresis cycle and qualitatively investigated by visualization of the colloid under exposure to a 0.4 T magnet. Gemcitabine entrapment into the polymeric shell reported adequate drug loading values (≈11%), and a biphasic and pH-responsive drug release profile (≈four-fold faster Gemcitabine release at pH 5.0 compared to pH 7.4). Cytotoxicity studies in MCF-7 human breast cancer cells proved that the half maximal inhibitory concentration of Gem-loaded nanocomposites was ≈two-fold less than that of the free drug. Therefore, these core/shell nanoparticles could have great possibilities as a magnetically targeted Gemcitabine delivery system for breast cancer treatment.

## 1. Introduction

Gemcitabine (Gem) (molecular weight, M_W_: 299.66 g/mol; water solubility: 22.3 mg/mL; *n*-octanol-water partition coefficient (log *P*_OW_): 0.14) is an effective drug against numerous malignancies. In fact, it was approved for the first-line treatment of pancreatic, bladder, breast, and small-cell lung cancers [1]. This chemotherapy agent has exhibited a significant antitumour activity when given alone (single agent) or in combination regimens to treat breast cancer [2,3]. As a cytosine analogue, it causes cytotoxicity mainly through inhibition of deoxyribonucleic acid (DNA) synthesis and incorporation into ribonucleic acid (RNA) [4,5,6]. Given its hydrophilic nature, Gem requires active transport to cross the cell (plasma) membrane.

The main limitations of this anticancer drug include: (*i*) rapid metabolism into an inactive molecule, and (*ii*) a very short half-life (<20 min in human plasma) [1,5,6]. They create the need to use high doses, resulting in acute drug toxicity. Several investigations have been conducted, postulating the postulation of nanoparticle (NP)-based delivery systems to be a promising approach to optimize the pharmacokinetics and accumulation of Gem molecules into the tumour site, and to overcome chemoresistance [1,7,8,9,10,11,12].

Engineering of drug carriers has recently been implemented with the conceptualization of nanoparticulate systems with stimuli-triggered drug release properties [13,14,15], e.g., magnetic-, pH-, or heat-triggered drug release. In this scenario, superparamagnetic iron oxide nanoparticles (NPs) can produce significant outcomes given their magnetic drug targeting capacity to the cancer tissue, combined with their versatility as agents in both magnetic antitumor hyperthermia and magnetic resonance imaging [8,16,17,18,19,20]. These magnetic colloids are frequently surface functionalized, e.g., with polymers, to optimize their biocompatibility and safety [17,21], and drug delivery and targeting properties [15,17,22,23,24]. In this line, poly(*ε*-caprolactone) (PCL) is used to fabricate NP-based systems with interesting drug transport capacities, i.e., good drug loading (*DL*, %) and entrapment efficiency (*EE*, %) values and controlled drug release profiles [25,26,27]. Recently, pH-triggered drug release properties have been described for PCL-based NPs, when they are exposed to an acidic environment in the tumour interstitium or inside a lysosome [28,29].

In this work, the formulation of a core/shell nanoparticulate system was investigated, in which magnetite (Fe_3_O_4_) nuclei are embedded into a PCL matrix containing Gem molecules. A complete physicochemical characterization of the nanocomposites was completed to evaluate the reproducibility and efficacy of the preparation procedure. Gem loading to the NPs and in vitro drug release profiles have been evaluated by spectrophotometry, while magnetic evaluations helped in analysing the magnetic responsiveness. Finally, the Fe_3_O_4_/PCL NPs were examined for their cytotoxicity against breast cancer cells. Despite this colloidal system having been postulated for the delivery of Gem molecules to malignant cells [30,31], as far as we know, this is the first time that Gem-loaded Fe_3_O_4_/PCL NPs have shown promising activity against breast cancer.

## 2. Materials and Methods

### 2.1. Materials

Poly(*ε*-caprolactone) (average M_W_ ≈ 14,000 determined by gel permeation chromatography) and Gemcitabine hydrochloride (C_9_H_12_ClF_2_N_3_O_4_) were obtained from Merck KGaA (Gernsheim, Germany). All other chemicals were of analytical grade from Panreac (Barcelona, Spain), except for Kolliphor^®^ P-188 (BASF, Ludwigshafen, Germany) and dichloromethane (Scharlab, S.L., Barcelona, Spain). Water used was previously deionized and filtered (Milli-Q Academic System, Millipore, Madrid, Spain).

### 2.2. Methods

#### 2.2.1. Preparation of Fe_3_O_4_/PCL (Core/Shell) NPs

**Figure 1:**
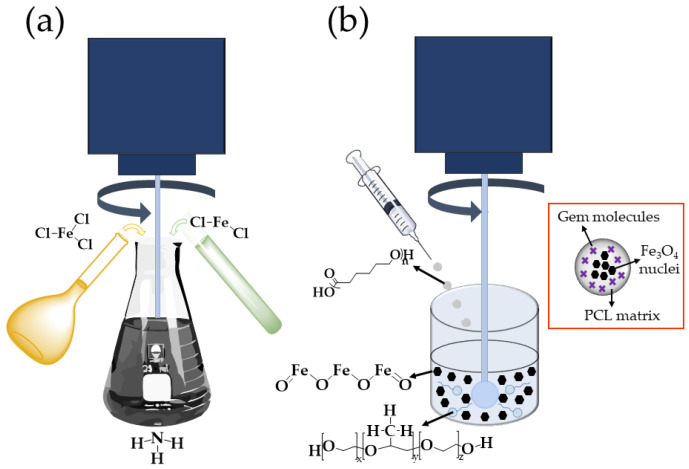
(**a**) Chemical co-precipitation to prepare Fe_3_O_4_ nanoparticle (NPs) [32], and, (**b**) formulation of the core/shell NPs by interfacial polymer disposition [25,27]. Inset: structure of the Gemcitabine (Gem)-loaded Fe_3_O_4_/poly(*ε*-caprolactone) (PCL) (core/shell) NPs.

Fe_3_O_4_ nuclei were obtained by chemical co-precipitation (Figure 1a) [32]. The method started with the simultaneous and slow addition of 40 mL of a 1 M FeCl_3_ solution and 10 mL of a 2 M FeCl_2_ solution (in 2 M HCl), to 0.5 L of a 0.7 M NH_3_ solution, at room temperature and under mechanical stirring (630 rpm; IKA^®^ Eurostar 60 Digital Constant-Speed Mixer, IKA, Königswinter, Germany). Long-term stabilization of these NPs was then completed by magnetic isolation (permanent magnet of 0.4 T) from the NH_3_ media and re-dispersion in 0.5 L of a 2 M HClO_4_ solution. After 12 h of contact, the magnetic cores were cleaned by repeated cycles of centrifugation (40 min at 9000 rpm, centrifuge 5804; Eppendorf Ibérica S.L.U., Madrid, Spain) until the conductivity of the supernatant was ≤10 μS/cm.

The magnetically responsive PCL colloid was prepared by an interfacial polymer disposition method (Figure 1b), which was also used to obtain pure PCL NPs [25,27]: 5 mL of a 1.25% (*w*/*v*) solution of PCL in dichloromethane were added, under mechanical stirring (1200 rpm), to 12.5 mL of a 2% (*w*/*v*) aqueous solution of Kolliphor^®^ P-188 containing the Fe_3_O_4_ nuclei (0.125%, *w*/*v*) (natural pH ≈ 5.8). Then, mechanical stirring (1200 rpm) of the colloidal suspension continued for 15 min, and the remaining organic solvent was evaporated in a rotary evaporator (Rotavapor^®^ R II, Büchi, Flawil, Switzerland) to obtain an aqueous dispersion of the magnetically responsive core/shell nanostructures. Finally, the Fe_3_O_4_/PCL particles underwent a cleaning procedure consisting of their repeated separation from the aqueous medium by using a permanent magnet (0.4 T) and re-dispersion in water until the conductivity of the supernatant was ≤10 μS/cm.

To determine the influence of the relative amounts of PCL and Fe_3_O_4_ on the properties of the resulting Fe_3_O_4_/PCL particles, the formulation was repeated with Fe_3_O_4_:PCL proportions ranging from 1:4 to 4:1. All experiments were carried out in triplicate (*n* = 3). The NPs production yield or performance (%) under all of these Fe_3_O_4_:PCL proportions was further determined (Equation (1)):

Yield (%) = [amount of Fe_3_O_4_/PCL NPs obtained (mg)/summation of materials used in the preparation of these NPs (mg)] × 100, (1)

Gem-loaded NPs were obtained by dissolving the chemotherapy agent in the aqueous phase, at a given amount (up to 1 mM), before incorporation of the dichloromethane phase.

#### 2.2.2. Characterization

Particle size, size distribution (polydispersity index, PdI), and zeta potential (*ζ*) were determined after appropriate dilution of the colloidal formulations in water (≈0.1%, *w*/*v*) and sonication for 0.5 min at 20% output (Branson Sonifier 450, Emerson Electric Co., St. Louis, MO, USA) (*n* = 3) (Zetasizer Nano-ZS, Malvern Instruments Ltd., Malvern, UK). Cell temperature was 25.0 ± 0.5 °C, and the detection angle was 90°.

High resolution transmission electron microscopy (HRTEM), high-angle annular dark field scanning transmission electron microscopy (HAADF-STEM), and annular bright field scanning transmission electron microscopy (ABF-STEM) (Titan G2 60-300 FEI microscope, Thermofisher Scientific™, Waltham, MA, USA) were used to visualize the core/shell nanostructure and to evaluate the complete coating of the iron oxide cores by the polymeric matrix. Drops of the dilute NPs dispersion (≈0.1%, *w*/*v*) were poured on formvar/carbon-coated copper microgrids, and dried in a convection oven (25.0 ± 0.5 °C, 24 h) (J.P. Selecta, S.A., Barcelona, Spain). Elemental analysis was performed during the HRTEM measurements by using an energy dispersive X-ray (EDX) spectrometer (Bruker Nano GmbH, Berlin, Germany).

The presence of the PCL coating onto the Fe_3_O_4_ nuclei was qualitatively evaluated by analysing the influence of the KNO_3_ concentration (at a constant pH ≈ 6) on the *ζ* values of the colloids. Determinations were done at room temperature (*n* = 9), after 12 h of contact under mechanical stirring (150 rpm, universal orbital shaker OS-10, Boeco, Hamburg, Germany). Electrokinetic measurements in water (pH ≈ 6) were also used for checking the properties of the polymer coating onto the magnetic cores when the Fe_3_O_4_:PCL ratios ranged from 1:4 to 4:1.

Chemical characterization of Fe_3_O_4_, PCL, and Fe_3_O_4_/PCL NPs was accomplished by Fourier transform infrared (FTIR) spectrometry (FT/IR-6200 spectrometer, JASCO, Portland, OR, USA; resolution of 0.25 cm^−1^). Significant bands of the nanomaterials were identified by comparison with published data [33,34].

The model developed by van Oss [35] was used to perform the surface thermodynamic analysis of the nanoformulations, which is capable of evaluating the surface-free energy components of a solid (*γ_S_*). It has been demonstrated to be exceptionally appropriate in the characterization of pharmaceutical colloids [17,36] and suspensions [37,38]. More details on the model can be found in the literature. The advancing contact angles (*θ*) of water, formamide, and diiodomethane were measured on dry NP layers (Ramé-Hart 100-00 telegoniometer, Ramé-Hart, Succasunna, NJ, USA) at 25.0 ± 0.1 °C (*n* = 12), inside a thermostatic chamber. Images of these drops softly deposited onto the layers (≈4 µL, Gilmont micrometer syringe, Gilmont, Gilmer, TX, USA) were captured with a video camera adapted to the telegoniometer.

#### 2.2.3. In Vitro Determination of Gemcitabine Loading and Release

Quantification of the anticancer agent incorporated to the magnetopolymeric nanosystem was conducted by spectrophotometric determinations of Gem molecules remaining in the supernatant after NP centrifugation (45 min, 9000 rpm) (Thermo Scientific™ Sorvall™ Legend™ Micro 21R microcentrifuge, ThermoFisher Scientific, USA) (*n* = 3), which was inferred from the total amount of drug in these aqueous dispersions. In this evaluation, the contribution to the absorbance of sources other than variations in Gem concentration was taken into account (e.g., Kolliphor^®^ P-188) by subtracting the absorbance of the supernatant produced under the same conditions but without the drug.

These UV absorption measurements were performed at the maximum absorbance wavelength (269 nm) (8500 UV-Vis Dinko spectrophotometer, Dinko, Barcelona, Spain). Good linearity was observed at this wavelength, and the method was previously validated and verified for accuracy, precision, and linearity. Gem incorporation to the Fe_3_O_4_/PCL particles was calculated in terms of *EE* (%) (Equation (2)), and *DL* (%) (Equation (3)):

*EE* (%) = [entrapped Gem (mg)/total Gem used in the experiments (mg)] × 100, (2)

*DL* (%) = [entrapped Gem (mg)/Gem-loaded NPs (mg)] × 100, (3)

Drug release experiments were accomplished using the Fe_3_O_4_/PCL NPs with the higher Gem loadings (i.e., *EE* ≈ 88% and *DL* ≈ 11%, see Table 1), following the dialysis bag method (*n* = 3), and at 37.0 ± 0.5 °C. The release media were either citrate-phosphate buffer reproducing the pH of bloodstream (7.4 ± 0.1), or the acidic microenvironment in tumours (pH 5.0 ± 0.1). Before use, the dialysis bags (cut-off of 2000 Da, Spectrum^®^ Spectra/Por^®^ 6 dialysis membrane tubing, Spectrum, New York, NY, USA) were soaked in water at room temperature for 12 h. Then, 2 mL of the dispersion of Gem-loaded NPs (containing 2 mg/mL of drug) was poured into the dialysis bag with the two ends fixed by clamps. The bags were placed in a glass beaker containing 150 mL of release media and stirred at 100 rpm. At prefixed time intervals (up to 14 days), 1 mL of the medium was withdrawn for UV spectrophotometric analysis of the drug content (269 nm). An equal volume of the release media, also kept at 37.0 ± 0.5 °C, was added after sampling to ensure the sink conditions. The same analytical procedure used to quantify the Gem loading was used.

#### 2.2.4. In Vitro Proliferation Studies

Cytotoxicity of blank (Gem unloaded) magnetic PCL particles was investigated in the CCD-18 human colon fibroblast cell line (Scientific Instrumentation Centre, University of Granada, Granada, Spain), and in the MCF-7 human breast adenocarcinoma cell line (American Type Culture Collection, ATCC, Manassas, VA, USA). Triplicate cultures were evaluated by the 3-(4,5-dimethylthiazol-2-yl)-3,5-diphenyl tetrazolium bromide (MTT) proliferation assay. Detailed description of the procedure can be found in the literature [39]. Cell lines were kept in contact with NP concentrations, ranging from 0.05 to 100 μg/mL, for 48 and 72 h.

The MTT assay was also used to define the cytotoxicity against MCF-7 cells of the Gem-loaded Fe_3_O_4_/PCL particles (of 2:4 weight ratio) in comparison with free Gem (*n* = 3). The concentration of these nanoformulations in contact with cells ranged from 0.1 to 50 µM equivalent Gem concentrations.

Cells without treatment were used as a control to calculate the relative cell viability (%) (Equation (4)).

Relative cell viability (%) = [optical density of treated cells/optical density of control (untreated) cells] × 100,(4)

#### 2.2.5. Magnetic Field Responsive Behaviour

Magnetic properties of the Fe_3_O_4_/PCL particles (of 2:4 weight ratio) were characterized at 25.0 ± 0.5 °C (Manics DSM-8 vibrating magnetometer, France). The magnetic field responsive behaviour of a 0.5% (*w*/*v*) aqueous dispersion was qualitatively analysed by optical visualization using a 0.4 T permanent magnet close to the glass vial containing the colloid. Optical microscope visualization of the colloid under exposure to this permanent magnet was possible by using an Olympus BX40 stereoscopic zoom microscope (Olympus, Tokyo, Japan).

#### 2.2.6. Statistical Analysis

Statistical analyses were conducted using the IBM SPSS Statistics software package (version 26.0; IBM Corporation, Armonk, NY, USA). Experimental data are expressed as means ± standard deviations. Student’s *t*-test helped to compare results considering 95% confidence interval. Differences were considered statistically significant at *p* < 0.05.

## 3. Results and Discussion

### 3.1. Characterization of the Fe_3_O_4_/PCL Nanocomposites

Biological fate and the use of magnetic nanosystems in Biomedicine are strongly influenced by their physical, chemical and physicochemical properties, e.g., size, shape, surface electrical charge, and surface thermodynamics [17].

**Figure 2:**
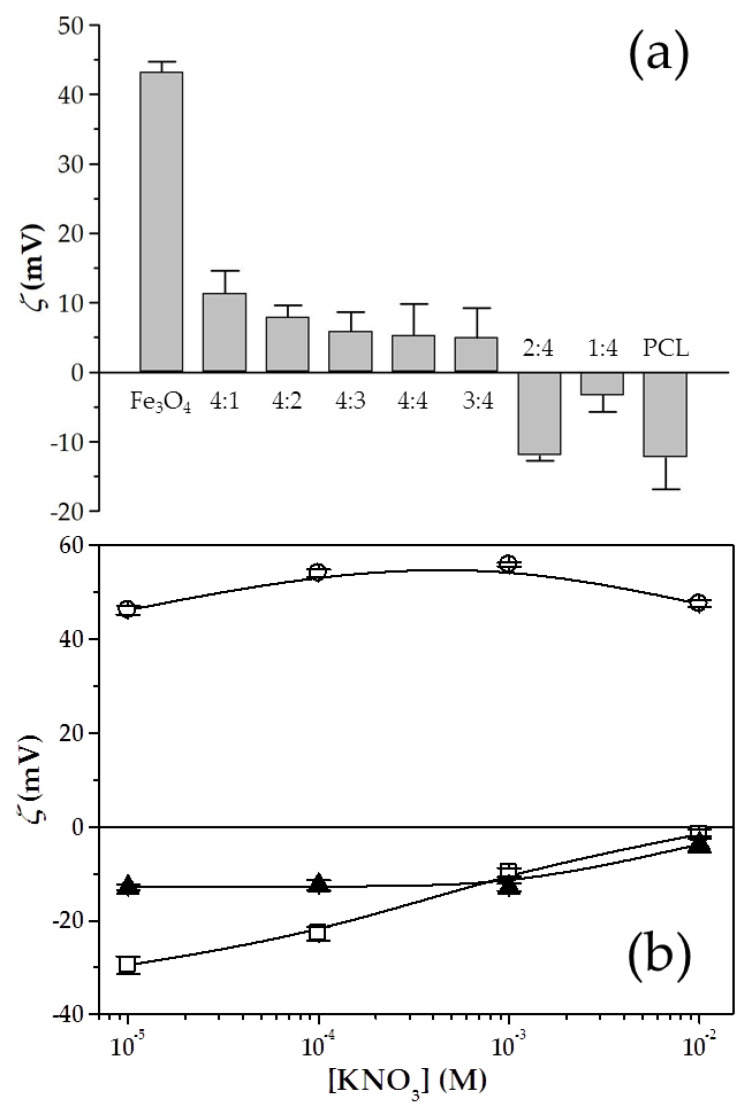
(**a**) Zeta potential (*ζ*, mV) of the nanocomposites of Fe_3_O_4_:PCL weight proportions ranging from 4:1 to 1:4, Fe_3_O_4_ NPs, and PCL NPs, in water (pH ≈ 6), and (**b**) zeta potential (*ζ*, mV) of Fe_3_O_4_ NPs (○), PCL NPs (□), and Fe_3_O_4_/PCL NPs (▲, of 2:4 Fe_3_O_4_:PCL weight ratio) as a function of the molar concentration of KNO_3_ at pH ≈ 6. Lines are a guide to the eye and have no other significance.

Mean diameter of Fe_3_O_4_ nuclei and pure PCL NPs was found to be 10 ± 3 and 145 ± 3 nm, respectively. The effect of the Fe_3_O_4_:PCL weight proportion on some of the relevant characteristics of the magnetopolymer composite particles, i.e., size, surface charge, and production performance, is summarized in Figure 2a and Table 1. When the mass of magnetic nuclei exceeded the amount of PCL used in the formulation or when the mass of polymer used was slightly greater than that of Fe_3_O_4_, positive surface electrical charges were characteristic for the Fe_3_O_4_/PCL NPs. This could be the consequence of the inadequate PCL coverage of the Fe_3_O_4_ cores. On the contrary, when the PCL quantity in the nanoformulations was increased, at very high ratios (≥2:4 Fe_3_O_4_:PCL), the complete surface coating of the Fe_3_O_4_ nuclei occurred and the surface electrical charge was found to be negative, just like the polymer. No relevant influence on particle size was found when changing the Fe_3_O_4_:PCL ratios, except that size and PdI was notably increased at the 1:4 proportion. Such a behaviour has been previously described for PCL-based systems, in which high polymer concentrations determine an increase in particle size [40,41]. It is suggested that as the polymer mass added to the organic phase is increased, the viscosity rises and this may render it more resistant to the shear forces which restricts the formation of the particles. Regarding the reduction in the zeta values observed for the NPs of 2:4 Fe_3_O_4_:PCL weight ratio, it has been described how small sizes are easily affected by the random movement of fluid flow and other particles. As a consequence, the absolute value of *ζ* of small particles is greater than that of large particles [42]. This effect has been previously observed by our research group in nanosystems engineered for biomedical applications [43,44].

Finally, the yield (production performance, %) notably augmented when the PCL:Fe_3_O_4_ increased, possibly the result of a more efficient coverage of the nanocores. Taking into consideration this valuable information, it was concluded that the 2:4 weight proportion assured appropriate production performances (≈90%), and adequate (small) size and *ζ* values (≈125 nm and ≈−12 mV, respectively) that may delay recognition and clearance by the mononuclear phagocyte system [45]. As a consequence, these 2:4 nanocomposites would be characterized by prolonged plasma half-lives and significant accumulation into the tumour interstitium [17,46]. Hence, this was the weight ratio selected for the formulation of the Fe_3_O_4_/PCL NPs.

The significant differences (*p* < 0.05) between the electrophoretic characteristics of the magnetic nuclei and the PCL particles, shown in Figure 2b, justified the use of *ζ* determinations as a function of ionic strength (KNO_3_ molar concentration) for qualitatively checking the efficiency of the preparation procedure developed to obtain the magnetopolymer nanocomposite. The relevant similarities between the *ζ* values of the PCL particles and the Fe_3_O_4_/PCL NPs were identified, and also the differences in their electrokinetics from those of the Fe_3_O_4_ nuclei (*p* < 0.05). Little dependence of the electrokinetics of the magnetic nuclei on the ionic strength of the media was found, as previously reported [8,47,48]. Oppositely, a decrease in the absolute values of *ζ* of both PCL-based colloids occurred when the ionic strength was increased. That effect could be the consequence of the typical double-layer compression mechanism [47,48]. The positive surface electrical charge of the Fe_3_O_4_ particles may come from the amphoteric thin oxide layer formed onto their surface in oxidizing environments [43], while the negative surface electrical charge of the PCL-based NPs may result from the dissociation of the free acrylic groups (existing in the chemical structure) in water (pH ≈ 6) [49]. From an electrophoretic point of view, Fe_3_O_4_/PCL NPs are quite close to pure PCL particles.

Considering the data, the mechanism of formation of the nanocomposites could be hypothesized. Formation of the core/shell NPs took place in acidic conditions, when the organic solution of PCL was added to the aqueous dispersion of iron oxide nuclei (natural pH ≈ 5.8, see Section 2.2.1). Under these conditions, attractive electrostatic interactions were expected to occur between the positively charged Fe_3_O_4_ particles and the negatively charged polymer. As a consequence, the PCL matrix will tend to concentrate in the vicinity of the iron oxide surface, leading to the formation of the nanocomposites.

**Figure 3:**
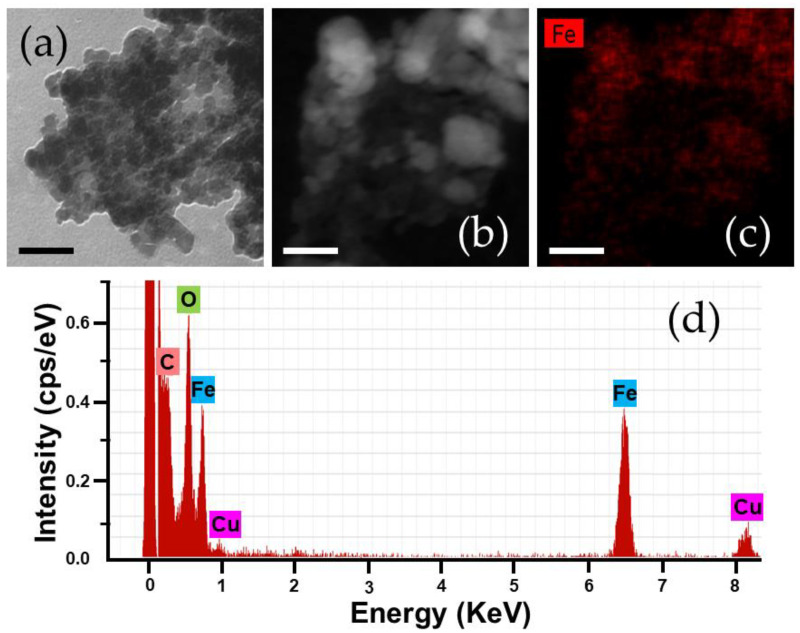
(**a**) High resolution transmission electron microscopy (HRTEM) and (**b**) high-angle annular dark field scanning transmission electron microscopy (HAADF-STEM) images of the magnetic nanocomposites (of 2:4 Fe_3_O_4_:PCL weight ratio); (**c**) energy dispersive X-ray (EDX) Fe element mapping analysis of the sample in (**b**), and (**d**) EDX spectra of these particles. Bar lengths: 50 nm.

HRTEM analysis of the Fe_3_O_4_/PCL NPs postulated the spherical shape of the nanocomposites (Figure 3a), in spite of particle aggregation. Aggregation of iron oxide/polymer (core/shell) NPs during sample preparation for EM observation has been previously described in magnetic poly(alkylcyanoacrylate)-based [50,51,52], and chitosan-based [36,53] nanocomposites. It has been associated with the method of sample preparation (drying), and was probably favoured by the hydrophobic PCL matrix in which the iron oxide nuclei were embedded.

HAADF-STEM photographs ratified that the iron oxide nuclei were satisfactorily embedded into the PCL matrix (Figure 3b), while the EDX Fe element mapping of the NPs demonstrated the homogeneous distribution of Fe within the PCL matrix (Figure 3c). EDX analyses also revealed the existence of the elements Fe, C, and O for the composite NPs (Figure 3d), of which the Fe element arises from the Fe_3_O_4_ cores [54,55], thus qualitatively confirming the incorporation of the Fe_3_O_4_ nanocores into the nanopolymer. The use of copper-based grids for EM characterization determined the appearance of the Cu element in this analysis.

**Figure 4:**
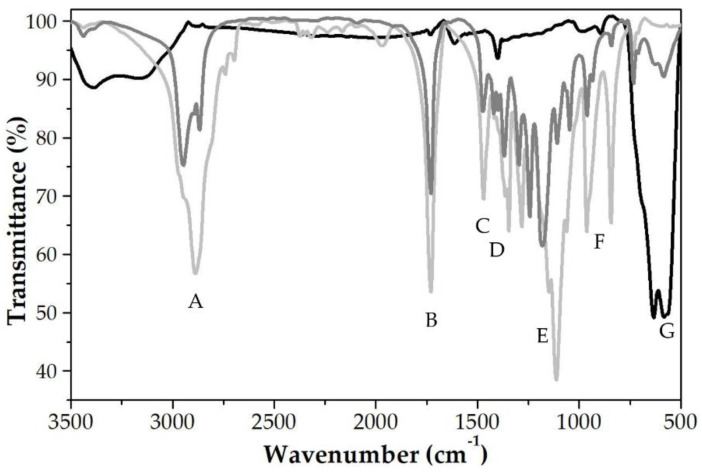
Infrared spectra of Fe_3_O_4_ NPs (black line), PCL NPs (light grey line), and nanocomposites (dark grey line, of 2:4 Fe_3_O_4_:PCL weight ratio).

Figure 4 collects the infrared spectra of the Fe_3_O_4_, pure PCL, and Fe_3_O_4_/PCL NPs. All the bands of the polymer were present in the spectrum of the magnetopolymer particles, therefore demonstrating that the shell observed in Figure 3a corresponded well to the PCL shell. Chemical groups identified in the spectra were: (A) C–H bond stretching vibration (at ≈2900 cm^−1^); (B) carbonyl stretching C=O of a carboxylic acid (at ≈1730 cm^−1^); (C) asymmetric CH_2_ bending vibration (at ≈1470 cm^−1^); (D) O–H bending vibration (at ≈1350 cm^−1^); (E) C-O stretching absorption at ≈ 1270 cm^−1^; (F) C–CO–C stretching and bending (at ≈1100 cm^−1^); (G) medium band characteristic of alkanes (at ≈950 cm^−1^); (H) CH rocking vibration characteristic of –CH long chains (at ≈830 cm^−1^), and (I) characteristic band of pure iron oxide NPs, not displayed by the pure PCL particles (at ≈600 cm^−1^).

**Figure 5:**
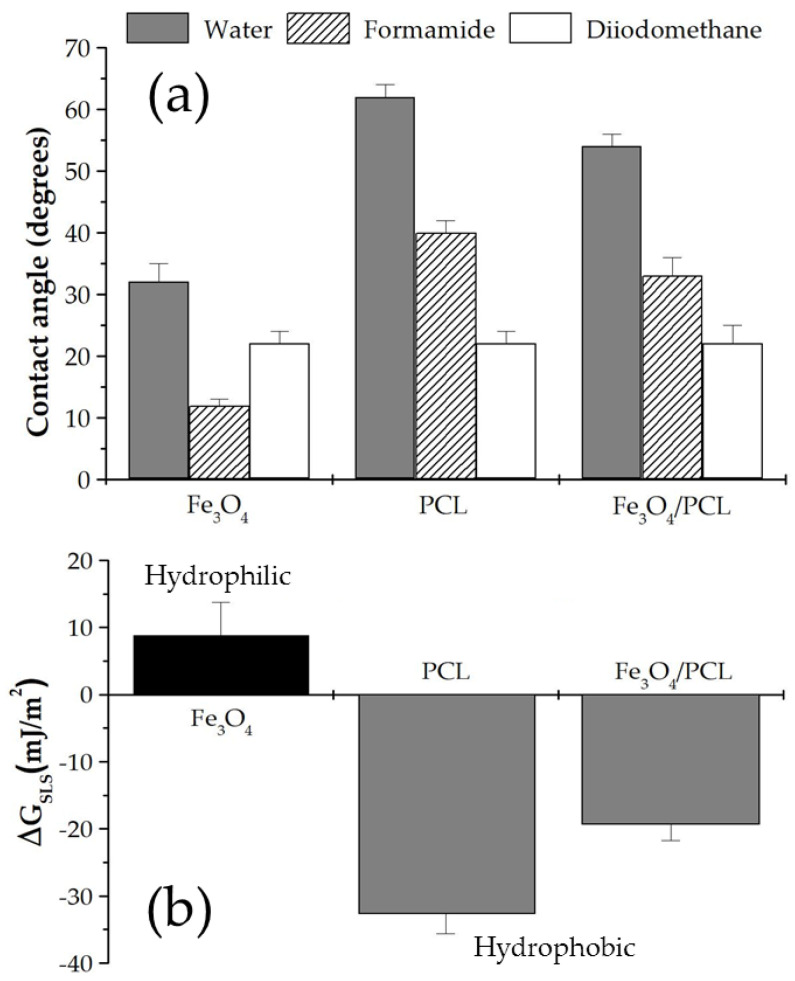
(**a**) Contact angle (θ, degrees) of water, formamide, and diiodomethane on Fe_3_O_4_, PCL, and Fe_3_O_4_/PCL (of 2:4 Fe_3_O_4_:PCL weight ratio) particle layers, and (**b**) Δ*G*_SLS_ (solid–liquid interfacial energy of interaction) values (mJ/m^2^) and hydrophobic/hydrophilic character of the NPs.

Analysis of the surface thermodynamics of the NPs started with the determination of *θ* of three liquids on dry sheets (Figure 5a). The results revealed significant differences among the three nanosystems investigated (Fe_3_O_4_, PCL, and Fe_3_O_4_/PCL) (*p* < 0.05), whereas the determination of the *γ_S_* reported a better characterization of the particle surface thermodynamics (Table 2). For instance, $\gamma_{S}^{-}$ presented large values in the iron oxide cores (a monopolar electron-donor material) than that existing for either the PCL NPs or the nanocomposites. These surface free energy changes were correlated with the hydrophilic/hydrophobic character of the NPs. The evaluation of the free energy of interaction between the solids immersed in the liquid (ΔGSLS) was used to establish if the colloid could be considered hydrophilic or hydrophobic in nature. This parameter is negative for hydrophobic solids, where interfacial interactions will favour attraction between the NPs. Oppositely, the hydrophilic character is related to positive values of ΔGSLS [17,35]. It was determined that the hydrophilic nature of Fe_3_O_4_ particles became hydrophobic, just like the PCL NPs, when covered by the polymeric shell (Figure 5b) (*p* < 0.05). Therefore, the thermodynamic analysis proved that the iron oxide nuclei were adequately embedded into the PCL matrix to form the nanocomposites.

### 3.2. Characterization Gemcitabine Absorption and In Vitro Release

The hydrophilic character of Gem was expected to determine a poor partition of the drug molecules from the aqueous phase to the hydrophobic matrix of PCL [10]. Thus, preparation conditions were established to minimize the escape of the chemotherapy agent from a mechanical trapping inside the magnetic nanocomposites. Firstly, how immediately just after the beginning of the interfacial polymer disposition method, the PCL nanomatrix precipitated when contacting the aqueous phase was checked. This may lead to an easier mechanical trapping of the drug within the PCL matrix [25]. Additionally, the stabilizing agent (Kolliphor^®^ P-188) may facilitate drug inclusion into the polymeric network, as it may induce the opening of the polymer chains to create a space within the PCL matrix where Gem can be loaded [25,56]. More importantly, Gem loading to the polymer matrix may be favoured by electrostatic attractions between the negatively charged PCL (≈−20 mV at natural pH 6) and the positively charged Gem molecules. Protonation of the –NH group of the drug molecule may generate the positively charged species facilitating this entrapment effect. Oppositely, Gem incorporation onto the surface of the iron oxide nuclei of the nanocomposites is not expected to take place, given the electrostatic repulsion that may occur between the positively charged drug and the positively charged Fe_3_O_4_ (≈+43 mV at natural pH 6). Table 3 collects the *EE* (%) and *DL* (%) data as a function of the Gem concentration. The concentration of antitumour drug positively influenced the Gem absorption efficiency into the nanocomposites. Finally, size and *ζ* values of the magnetopolymeric NPs (of 2:4 Fe_3_O_4_:PCL weight ratio) did not vary significantly when loaded with Gem molecules: ≈130 nm and ≈−10 mV, respectively.

**Figure 6:**
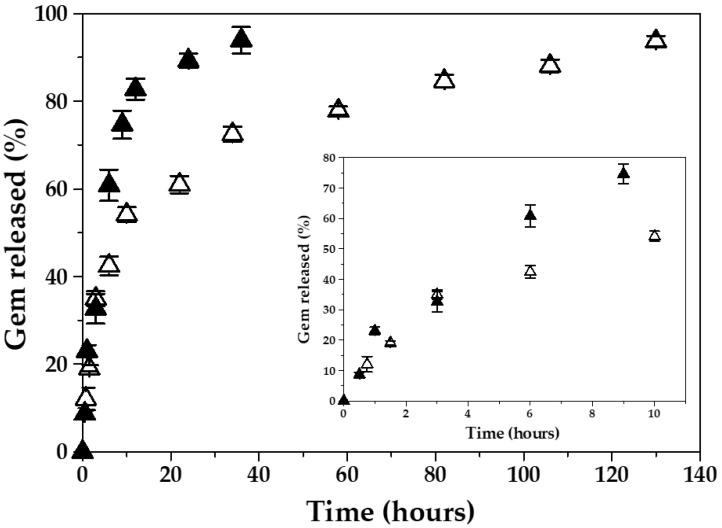
In vitro release of Gem (%) from the magnetic nanocomposites (of 2:4 Fe_3_O_4_:PCL weight ratio), as a function of the incubation time at 37.0 ± 0.5 °C, in citrate-phosphate buffers of 7.4 ± 0.1 (∆) and of pH 5.0 ± 0.1 (▲). Inset: Gem released (%) up to *t* = 10 h.

Rapid blood metabolization of Gem is likely to be one of the major factors limiting its clinical success [1]. Loading of Gem molecules inside a biodegradable nanocarrier may contribute to the optimization of the pharmacokinetics and accumulation of the antitumour drug into the tumour site [28]. To that aim, the nanoparticulate system is expected to minimize drug release in the blood (pH ≈ 7.4), while facilitating a rapid (burst) release of the anticancer agent when reaching the tumour interstitium (pH ≈ 5). This may assure the deep contact of cancer cells with the antitumour molecules. In order to check if the Fe_3_O_4_/PCL (core/shell) NPs displayed a pH-responsive Gem release, the release media reproduced either the pH 7.4 of bloodstream or the acidic microenvironment in tumours (pH 5) (Figure 6). A biphasic process was observed, which is characteristic of this polymeric entity [25,57,58]. The process started with an early rapid drug release, taking place within 6 h (up to ≈40% at pH 7.4, and ≈60% at pH 5.0), with the remaining Gem molecules being slowly liberated during the next 124 h at pH 7.4, and 30 h at pH 5.0. The rapid first phase release may result from the leakage of the surface-associated and/or poorly entrapped chemotherapy agent. Contrarily, Gem release during the slower release phase may result from drug diffusion through the PCL matrix, rather than from polymer degradation (a very slow process in water due to the semi-crystallinity and hydrophobic character of the polymer) [25,58]. Such a biphasic profile suggested that the major drug fraction was absorbed into the PCL shell rather than adsorbed onto the nanocomposite surface. Finally, the NPs showed a pH-responsive Gem release (≈four-fold faster Gem release at pH 5.0 compared to pH 7.4) (*p* < 0.05), a behaviour previously described for this polymeric matrix [29], which may be advantageous for the selective or complete intratumour delivery of Gem. The faster Gem release at the acidic pH could be associated to the higher rate of hydrolysis of the ester linkages in the PCL structure, compared to the normal blood pH 7.4 [28,29,59].

### 3.3. In Vitro Cytotoxicity

**Figure 7:**
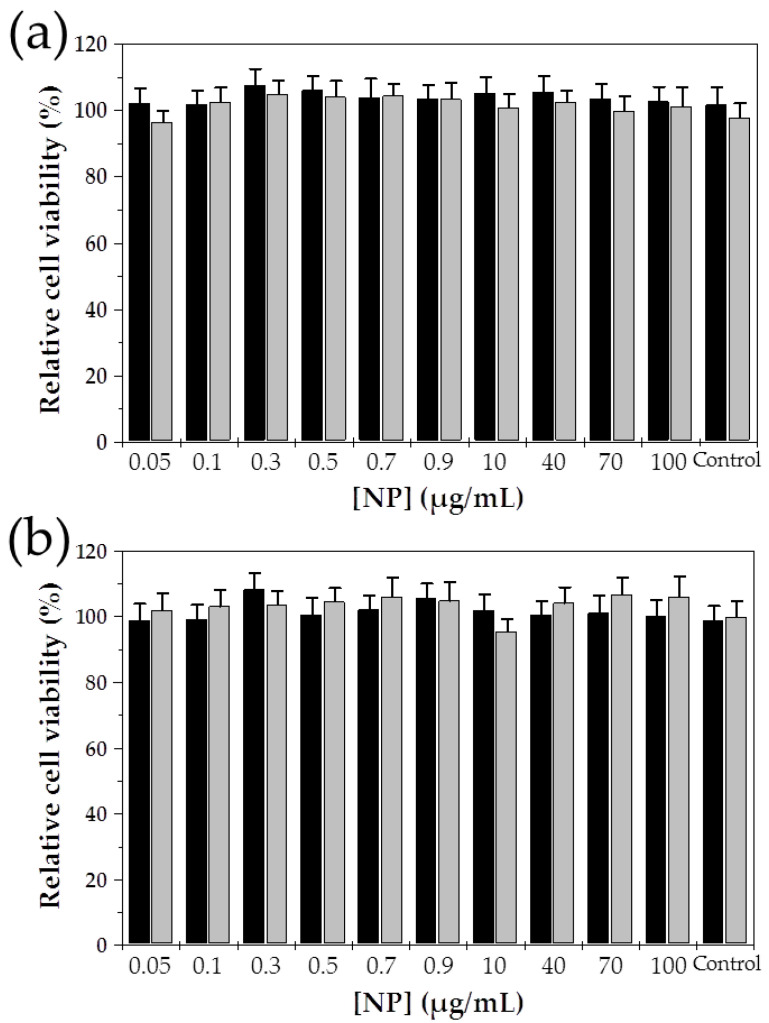
In vitro cytotoxicity of the Fe_3_O_4_/PCL NPs (of 2:4 Fe_3_O_4_:PCL weight ratio) in: (**a**) CCD-18 human colon fibroblast cells, and (**b**) MCF-7 human breast cancer cells. Cell lines were kept in contact with the particles for 48 h (black column) and 72 h (light grey column).

Blank (Gem-unloaded) Fe_3_O_4_/PCL NPs exhibited negligible cytotoxicity in normal CCD-18 and tumour MCF-7 human cell lines at the dilutions corresponding to those of the Gem-loaded magnetic nanocomposites (Figure 7). In fact, differences in cell growth between the control group and cells kept in contact with the particles were not determined to be statistically significant at any of the concentrations investigated. It has been described how the growth of cells kept in contact with non-cytotoxic blank (drug-unloaded) NPs is not hindered [60,61,62], even at high concentrations. As a result, proliferation can continue under in vitro conditions. Based on these results, they may present an adequate biocompatibility and safety for drug delivery purposes.

**Figure 8:**
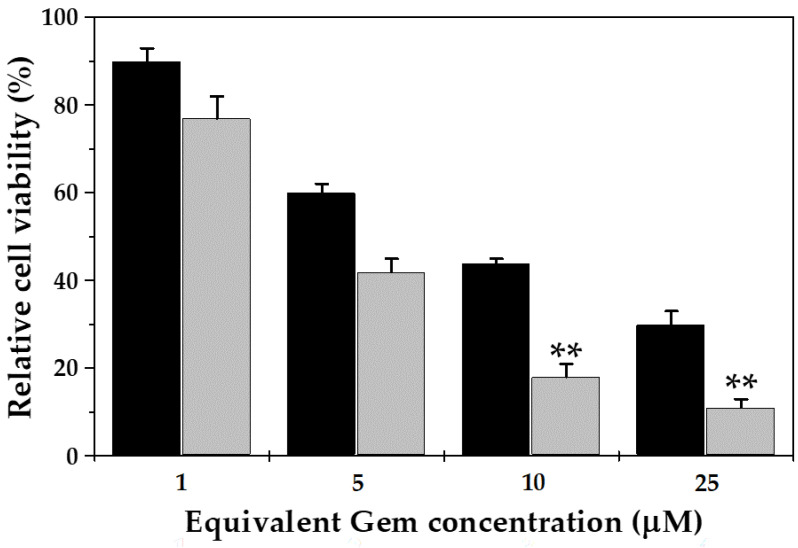
In vitro cytotoxicity of free Gem (black column) and Gem-loaded magnetic nanocomposites (of 2:4 Fe_3_O_4_:PCL weight ratio) (light grey column) in MCF-7 human breast cancer cells, after 72 h of exposure to a wide range of NP concentrations (up to 25 μM equivalent Gem concentration). Statistical analysis was done using Student’s *t*-test considering 95% confidence interval. The statistical test was significant, ** *p* < 0.05, compared with the free Gem-treated group.

The in vitro cytotoxic effect of free Gem and Gem-loaded Fe_3_O_4_/PCL NPs in MCF-7 cells is displayed in Figure 8. These Gem-based formulations inhibited cell proliferation in a dose-dependent manner. Compared to free Gem, the improved anticancer activity of Gem-loaded magnetic nanocomposites (of 2:4 Fe_3_O_4_:PCL weight ratio) was statistically significant at 10 and 25 μM equivalent drug concentrations (*p* < 0.05). In addition, the half maximal inhibitory concentration (IC_50_) value of the Gem-loaded NPs (4.27 ± 0.39 μM) was ≈two-fold less than that of free Gem (8.55 ± 0.31 μM) (*p* < 0.05). The higher cytotoxicity of the Gem-loaded nanocomposites compared to the free drug is in agreement with previous studies, in which Gem loading to a NP is hypothesized to facilitate uptake by cancer cells [10,11].

### 3.4. In Vitro Magnetic Responsiveness

**Figure 9:**
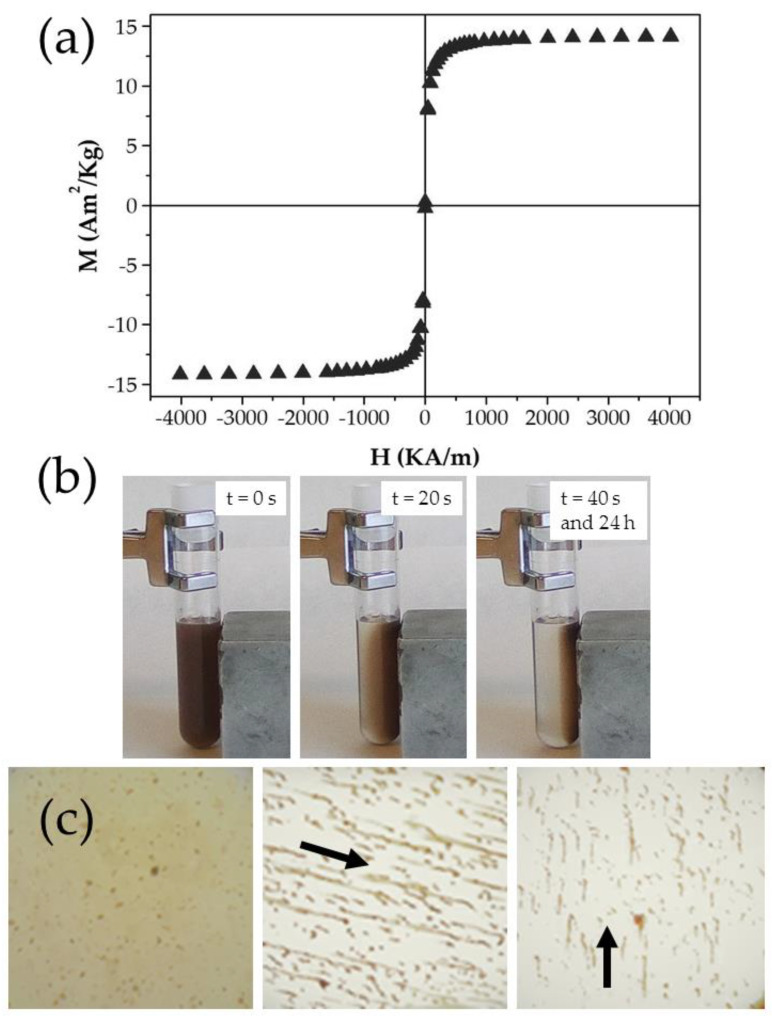
(**a**) Hysteresis cycle of the Fe_3_O_4_/PCL NPs (of 2:4 Fe_3_O_4_:PCL weight ratio, ▲); (**b**) visual observation of an aqueous dispersion (0.5%, *w*/*v*) of these particles on a 400 mT permanent magnet (located close to the right lateral flat surface of the glass vial), and (**c**) optical microphotographs of the aqueous colloids under the influence of this magnetic field (in the direction of the arrow).

The field-responsive behaviour of the Fe_3_O_4_/PCL NPs was investigated by checking their hysteresis cycle (Figure 9a): the nanocomposites apparently exhibited a soft magnetic character, given that the increasing and decreasing field ramps of the cycle were hardly discernible with the sensitivity of the instrument used. From the linear portions (low field) of the curve the initial susceptibility was calculated: (0.189 ± 0.032) × 10^−3^ m^3^/Kg; along with the saturation magnetization: 13.74 ± 0.97 Am^2^/Kg for the particles. The suitable magnetic responsive behaviour of the nanocomposites is further illustrated in Figure 9b,c. Complete magnetic attraction of the NPs towards the 400 mT magnet occurred in 40 s (Figure 9b). Magnetic responsiveness of the particles was also evaluated by optical microscopy inspection of the colloid under exposure to that magnetic field (Figure 9c): the initially homogeneous aqueous dispersion of NPs was intensely changed, and formation of chainlike aggregates parallel to the field lines was observed. This could be the consequence of the relevant contribution of the magnetic interaction over the DLVO colloidal interactions between NPs (e.g., electrostatic van der Waals and hydration or acid-base), despite the existence of the PCL shell. However, in vivo experiments should be done to perfectly define if this field-responsive behaviour could favour the concentration of nanocomposites into the tumour interstitium.

## 4. Conclusions

A reproducible and efficient methodology to formulate Fe_3_O_4_/PCL NPs (average size ≈ 125 nm, production performance ≈ 90%) has been developed. The large characterization completed, i.e., electron microscope and EDX analysis, FTIR spectroscopy, and evaluation of the electrophoretic and surface thermodynamic characteristics, confirmed the complete coverage of the iron oxide nuclei by the PCL shell. Negligible cytotoxicity and magnetic responsiveness of the nanocomposites were demonstrated in vitro. In addition, drug loading conditions reported an appropriate Gem incorporation by the Fe_3_O_4_/PCL particles (*EE* ≈ 88% and *DL* ≈ 11%), along with sustained (biphasic) and pH-responsive Gem release properties (≈four-fold faster Gem release at pH 5.0 compared to pH 7.4). Finally, Gem-loaded NPs exhibited a superior cytotoxicity over the free chemotherapy agent against MCF-7 breast cancer cells (IC_50_ ≈ two-fold less than that of free Gem). These core/shell nanostructures may constitute a potential candidate for breast cancer treatment.

## Figures and Tables

**Table 1:** Yield (%), and size (nm) and PdI of the Fe_3_O_4_/PCL NPs for the relative weight proportions of Fe_3_O_4_ and PCL ranging from 4:1 to 1:4.

Fe_3_O_4_:PCL Weight Ratio	4:1	4:2	4:3	4:4	3:4	2:4	1:4
Yield (%)	≈15	≈20	≈30	80.1 ± 7.6	69.1 ± 12.2	91.5 ± 8.1	57.9 ± 6.3
Size (nm)	136 ± 2	142 ± 3	138 ± 3	196 ± 16	278 ± 7	126 ± 1	455 ± 107
PdI	0.21 ± 0.02	0.23 ± 0.01	0.23 ± 0.02	0.41 ± 0.01	0.31 ± 0.01	0.26 ± 0.03	0.93 ± 0.06

**Table 2:** Surface free energy components ($\gamma_{S}$, mJ/m^2^) of the Fe_3_O_4_, PCL, and Fe_3_O_4_/PCL (of 2:4 Fe_3_O_4_:PCL weight ratio) particles. $\gamma_{S}^{LW}$, $\gamma_{S}^{+}$, and $\gamma_{S}^{-}$ are the Lifshitz-van der Waals, electron-acceptor, and electron-donor components, respectively.

Solid	$\gamma_{S}^{LW}$ (mJ/m^2^)	$\gamma_{S}^{+}$ (mJ/m^2^)	$\gamma_{S}^{-}$ (mJ/m^2^)
Fe_3_O_4_	47.17 ± 0.64	0.68 ± 0.15	37.75 ± 3.01
PCL	47.17 ± 0.64	0.16 ± 0.01	14.75 ± 1.23
Fe_3_O_4_/PCL	47.17 ± 0.96	0.33 ± 0.04	20.45 ± 0.91

**Table 3:** Entrapment efficiency (*EE*, %) and loading (*DL*, %) of Gem to the Fe_3_O_4_/PCL NPs (of 2:4 Fe_3_O_4_:PCL weight ratio).

[Gem] (M)	EE (%)	DL (%)
10^−5^	12.232 ± 1.483	0.016 ± 0.012
5 × 10^−5^	31.286 ± 1.014	0.163 ± 0.013
10^−4^	56.007 ± 0.264	1.094 ± 0.185
5 × 10^−4^	71.013 ± 0.994	6.433 ± 1.141
10^−3^	87.468 ± 0.332	11.174 ± 3.222
